# Identification of wounding and topping responsive small RNAs in tobacco (*Nicotiana tabacum*)

**DOI:** 10.1186/1471-2229-12-28

**Published:** 2012-02-22

**Authors:** She Tang, Yu Wang, Zefeng Li, Yijie Gui, Bingguang Xiao, Jiahua Xie, Qian-Hao Zhu, Longjiang Fan

**Affiliations:** 1 Department of Agronomy and James D. Watson Institute of Genome Sciences, Zhejiang University, Hangzhou 310058, Zhejiang, China; 2 Yunnan Academy of Tobacco Agricultural Sciences and China Tobacco Breeding Research Center at Yunnan, Yuxi 653100, Yunnan, China; 3 Department of Pharmaceutical Sciences, North Carolina Central University, Durham, NC 27707, USA; 4 CSIRO Plant Industry, Canberra ACT 2601, Australia

## Abstract

**Background:**

MicroRNAs (miRNAs) and short interfering RNAs (siRNAs) are two major classes of small RNAs. They play important regulatory roles in plants and animals by regulating transcription, stability and/or translation of target genes in a sequence-complementary dependent manner. Over 4,000 miRNAs and several classes of siRNAs have been identified in plants, but in tobacco only computational prediction has been performed and no tobacco-specific miRNA has been experimentally identified. Wounding is believed to induce defensive response in tobacco, but the mechanism responsible for this response is yet to be uncovered.

**Results:**

To get insight into the role of small RNAs in damage-induced responses, we sequenced and analysed small RNA populations in roots and leaves from wounding or topping treated tobacco plants. In addition to confirmation of expression of 27 known miRNA families, we identified 59 novel tobacco-specific miRNA members of 38 families and a large number of loci generating phased 21- or 24-nt small RNAs (including ta-siRNAs). A number of miRNAs and phased small RNAs were found to be responsive to wounding or topping treatment. Targets of small RNAs were further surveyed by degradome sequencing.

**Conclusions:**

The expression changes of miRNAs and phased small RNAs responsive to wounding or topping and identification of defense related targets for these small RNAs suggest that the inducible defense response in tobacco might be controlled by pathways involving small RNAs.

## Background

Small RNAs are a group of regulatory molecules that fall into two major classes, microRNAs (miRNAs) and short interfering RNAs (siRNAs). They play important roles in biological systems in eukaryotes by suppressing expression of target genes at the transcriptional and/or post-transcriptional level through specific base pairing with their targets [1]. In plants, siRNAs are further classified into *trans-*acting siRNAs (ta-siRNAs), natural antisense transcript-derived siRNAs (nat-siRNAs), and repeat-associated siRNAs (ra-siRNAs) [2]. In addition, a novel class of bacteria-induced 30- to 40-nt endogenous small RNAs, long siRNAs (lsiRNAs), was identified in *Arabidopsis *[3].

As an important group of small RNAs, miRNA has attracted much attention. A number of studies have been performed to reveal the biogenesis of miRNAs and the mechanisms of miRNA-mediated gene regulation [4-6]. In plants, miRNA derives from primary miRNA transcript (pri-miRNA), which is transcribed by RNA polymerase II. After formation of a stem-loop secondary structure [7,8], pri-miRNA is cleaved twice by DICER-LIKE1 (DCL1), a RNase III enzyme [9]. The first cleavage of DCL1 releases a miRNA precursor (pre-miRNA), and the second cleavage of DCL1 generates a pair of miRNA/miRNA* duplex. The mature miRNA targets mRNA by being incorporated into the RNA-induced silencing complex (RISC) and plays a role in regulation of gene expression [4], while miRNA*, the complementary strand of the mature miRNA, is usually degraded and applies no function [10] although functional miRNA* has been reported [11]. In plants, miRNAs have been demonstrated to be involved in many biological and metabolic processes, including developmental regulation, growth control, cell differentiation, signal transduction, and biotic and abiotic stresses [5,12-15]. For example, miR172 regulates floral organ identity and flowering time by cleavage and/or translational repression of AP2-domain containing genes [16-18]; miR159 has been shown to be required for normal anther development by mediating the expression of GAMYB-related genes [19].

Unlike miRNAs, endogenous siRNAs arise from loci, in which double-stranded RNAs are able to be formed by the action of RNA-dependent polymerase or by two convergent transcripts. ta-siRNAs are 21-nt phased small RNAs and function like miRNAs. *TAS3 *is conserved in many plant species. In *Arabidopsis *and rice four and three *TAS *loci have been identified, respectively [20-22]. Besides ta-siRNAs, other types of phased small RNAs have also been reported recently, such as miRNA-like siRNAs from long hairpin [23] and phased small RNAs from superclusters flanked by a common 22-nt motif targeted by miR2118 [24].

As the major compounds of tobacco (*Nicotiana tabacum*), nicotine alkaloids have defensive functions in response to pathogen infection and herbivore wounding. Studies on alkaloids have been focused on the harm of smoking cigarettes and addictions to nicotine [25,26]. The potential use of nicotine alkaloids as insecticides in agriculture has also been investigated [27]. Nicotine alkaloids are synthesized in tobacco roots and delivered to leaves through the plant transport systems, consequently nicotine alkaloids are mainly accumulated in leaves in most *Nicotiana *species [28,29]. Nicotine biosynthesis can be induced through topping or leaf wounding to tobacco plants [30,31] and significant increase of nicotine content was observed in the wounded tobacco plants [32]. In the past 20 years, a series of genes involved in the nicotine biosynthetic pathways have been identified, e.g. *PMT *(putrescine *N*-methyltransferase) [33,34]; *MPO *(*N*-methylputrescine oxidase) [35]; *QPT *(quinolinate phosphoribosyltransferase) [36,37] and *A622 *[38]. However, it is unclear whether samll RNAs are responsive to wounding and are involved in induction of nicotines upon wounding.

Over 4,000 miRNAs (miRBase Release 16, http://www.mirbase.org) and several kinds of siRNAs have been identified in plants. Recently, identification of known conserved miRNAs has been reported in tobacco [39,40]. However, no tobacco-specific miRNA has been experimentally isolated. In this study, we intend to experimentally indentify miRNAs and siRNAs in the tobacco genome, especially small RNAs responsive to damages through high-throughput sequencing of small RNA populations from wounding or topping treated tobacco plants. To identify potential targets of small RNAs, a degradome from wounding treated plants was sequenced. As a result, 159 known and novel tobacco-specific miRNAs together with a number of loci generating phased small RNAs were identified. Expression levels of some of these small RNAs were changed after wounding or topping treatment, suggesting that these small RNAs may play a defensive role in response to wounding or topping damage in tobacco.

## Results

### Deep sequencing reveals a diverse set of tobacco small RNAs

To get insight into the composition of small RNA populations and their changes after damage treatments in tobacco, we isolated small RNAs from roots and leaves of leaf-wounding-treated plant, roots of topping-treated plant and roots of untreated plant. Small RNAs were then sequenced using the Illumina high-throughput sequencing technology. A total of 18.5 million of raw reads (15-35 nt) were generated from four samples (Table 1). Similar to previous reports on small RNA cloning in other plant species, 20-24-nt reads were dominated in all four small RNA populations with the 24-nt small RNAs the most abundant species (Figure 1). Of the 20-24-nt reads, 42.2% and 20.7% were perfectly mapped to the Tobacco Genome Initiative (TGI) GSS and EST datasets (see Methods). Many small RNAs were singletons or observed only in one sample. Less than 3% of the small RNA reads were common in all four samples. These results illustrate the complexity of the tobacco small RNA populations, which might partly be due to tetraploid characteristic of tobacco.

**Table 1:** Summary of high-throughput sequencing of tobacco small RNAs from root and leaf

Category	Root			Leaf
	
	Control	Topping	Wounding	Wounding
Total				
Raw reads	3,074,948	5,186,473	5,510,747	4,722,862
Unique raw reads	1,018,809	1,959,287	3,083,036	2,178,969
Mapped to GSS^#^				
Reads	1,277,575	2,632,084	2,700,532	2,469,534
Specific in each library	206,310	477,279	803,960	491,809
Conserved in all libraries	782,079	1,667,977	1,085,172	1,312,822
Unique	252,425	542,516	965,547	687,949
Specific in each libraries	148,981	385,469	702,522	442,337
Conserved in all libraries	25,780	25,780	25,780	25,780
Singleton	202,567	417,905	761,185	534,409
Mapped to EST^#^				
Reads	764,235	1,293,204	1,218,970	1,528,346
Specific in each libraries	43,758	99,553	137,509	107,459
Conserved in all libraries	563,565	985,513	811,452	1,165,580
Unique	72,750	129,710	186,601	149,454
Specific in each libraries	32,875	77,511	116,666	84,638
Conserved in all libraries	14,001	14,001	14,001	14,001
Singleton	48,486	88,566	131,515	102,406

#Only 19-25-nt reads were counted

**Figure 1:**
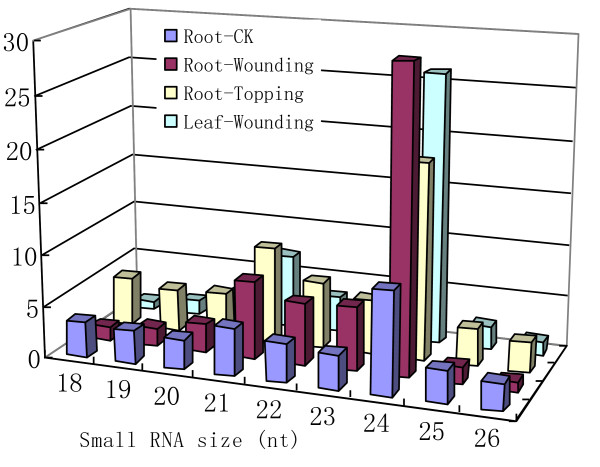
**Length distribution of small RNAs from different tissues and treatments**. Y-axis: number of normalized small RNA reads (×10^5^).

### Identification of miRNAs and loci generating phased small RNAs

#### miRNA identification

We searched miRBase for tobacco homologs of known miRNAs. As a result, homologs of 27 known miRNA families were found in our dataset. Based on BLAST search against the tobacco GSS and EST databases, we found that the mature miRNAs of these 27 known miRNA families match to 100 distinct GSS or EST sequences. All these sequences are able to form stem-loop structures; therefore they were considered as pre-miRNAs (Additional file 1: Table S1). Although 21 of these 27 miRNA families has been shown to be conserved in tobacco previously using a computational approach [31], our experimental approach confirmed the conservation of additional five miRNAs (miR408, miR477, miR1919, miR2118 and miR2911) in tobacco, which were not found computationally. Forty-four computationally identified tobacco miRNA families were not detected in our dataset, which could be because these miRNAs are lowly expressed and remain undetectable at the sequencing depth in our experiments or alternatively they are expressed in specific tissues that are not sampled in our experiments.

To identify novel tobacco miRNAs, we developed a miRNA discovery pipeline (see Methods). Using the custom-designed pipeline, we identified 59 novel tobacco miRNAs belonging to 38 families (Table 2; Additional file 1: Table S1). Of these 38 novel miRNA families, 20 families (53%) had a 21-nt mature miRNA sequences. Of these 20 families, 14 families (70%) had a mature miRNAs started with U. These results well supported the miRNA identity of these newly identified miRNAs. Of the 38 novel miRNA families, 13 families (34%) had a 22-nt mature miRNAs, which have been shown to be the secondary siRNA triggers [23,41]. Whether these 22-nt novel miRNAs play a role in the biogenesis of secondary siRNAs is waiting to be investigated. Twenty-seven of the 38 novel miRNA families contain only one member, suggesting their relatively recent origin.

**Table 2:** Novel tobacco miRNAs identified in this study

Novel miRNAs	Size (nt)	Pre-miRNA hit	Number of the mature miRNA reads (RPM)				Mature miRNA sequence
				
			Root			Leaf	
				
			Control	Topping	Wounding	Wounding	
Nta-miR1	21	ET738113.1	2.3	16.6	204.9	123.7	TGGCAACTTCTTCATCATGCC
Nta-miR2	21	FH134894.1	12.7	24.5	28.1	15.2	TCAATTGAGATGACATCTAGT
Nta-miR3	22	FH179115.1	12.7	20.6	2.4	1.9	CATTTTCACATGTAGCACTGAC
Nta-miR4a.1	22	ET742627.1	16.1	14.9	11.6	3.7	AACAATTGAGATAACATCTAGG
Nta-miR4b	22	FH277271.1	16.1	14.9	11.6	3.7	TGCCAACTATTGAGATGACATC
Nta-miR4a.2	22	ET742627.1	5.2	14.5	719.5	396.2	TGCCAACTATTGAGATGACATC
Nta-miR4c	22	ET817133.1	0.3	0.2	9.4	6.6	TGCCAATTATAGAGATGACATC
Nta-miR5a	22	FH599068.1	4.9	4.2	2.5	1.5	TTTGTCCAATGAAACACTTATC
Nta-miR5b	22	ET748842.1	70.9	45.9	21.0	25.6	TTTGTCCAATGAAATACTTATC
Nta-miR6	21	FH715144.1	1.6	3.5	4.7	5.3	TGACATCTTCAAAACCCACTA
Nta-miR7	21	ET849487.1	6.8	8.7	0.2	0.0	TACGTCGATCGATTGTTCTTA
Nta-miR8	21	ET878171.1	5.9	1.9	3.3	0.2	TGTGTTAATCGTTTGTTCTCA
Nta-miR9a	21	ET872202.1	14911.6	29389.3	2155.8	1094.8	TTGATACGCACCTGAATCGGC
Nta-miR9b	21	ET886878.1	14911.6	29389.3	2155.8	1094.8	TTGATACGCACCTGAATCGGC
Nta-miR10	22	FH007932.1	4.2	7.7	42.8	21.6	TACAGGTGACTTGTAAATGTTT
Nta-miR11	22	FH363222.1	2.9	3.9	1.3	1.5	AGATTTGTTTGATCGTCTTGGC
Nta-miR12	22	FH007932.1	4.6	1.9	224.3	171.1	AGATACTCAGCAAAACATTTAC
Nta-miR13a	22	ET680982.1	0.2	0.4	1.3	0.6	TGAATGTGAGGCATTGGATTGA
Nta-miR13b	22	ET781387.1	0.2	0.4	1.3	0.6	TGAATGTGAGGCATTGGATTGA
Nta-miR13c	22	ET788684.1	0.2	0.4	1.3	0.6	TGAATGTGAGGCATTGGATTGA
Nta-miR13d	22	ET967981.1	0.2	0.4	1.3	0.6	TGAATGTGAGGCATTGGATTGA
Nta-miR13e	22	FH063511.1	0.2	0.4	1.3	0.6	TGAATGTGAGGCATTGGATTGA
Nta-miR13f	22	ET676102.1	0.2	0.4	0.4	0.2	TGAGTGTGAGGCATTGGATTGA
Nta-miR13g	22	ET780880.1	4.9	9.4	1.5	1.6	TGAGTGTGAGGCGTTGGATTGA
Nta-miR13h	22	ET858637.1	4.9	9.4	1.5	1.6	TGAGTGTGAGGCGTTGGATTGA
Nta-miR13i	22	FH085512.1	4.9	9.4	1.5	1.6	TGAGTGTGAGGCGTTGGATTGA
Nta-miR14	21	ET964891.1	9.4	65.0	17.6	10.2	TGAGTGTGAGGCGTTGGATTGA
Nta-miR15a	24	ET740537.1	0.0	0.0	3.4	3.2	TATTGTATTCGACTGTATTCACGG
Nta-miR15b	24	FH095578.1	0.0	0.0	3.4	3.2	TATTGTATTCGACTGTATTCACGG
Nta-miR16	21	FH440616.1	2.3	0.8	5.6	1.9	TTATCATACGTAGCACTAGCC
Nta-miR17	21	FH731935.1	354.2	52.6	116.5	54.4	TAGGACCATATTCACTATTTG
Nta-miR18a	21	ET783551.1	3.3	1.2	24.2	23.1	TGGGTCTCCTGGAGAAAGGTC
Nta-miR18b	21	FH988094.1	3.3	1.2	24.2	23.1	TGGGTCTCCTGGAGAAAGGTC
Nta-miR19	21	ET839854.1	2.3	6.0	13.4	5.1	TAAGGTTGCCTTGCTCTTGCA
Nta-miR20	21	FH374163.1	2.9	1.2	23.8	11.9	AGTGGGTGGAGTGGTAAGATA
Nta-miR21	20	FH383824.1	0.0	0.2	10.0	1.7	CAGTGCACATATAACAGTAA
Nta-miR22	21	ET704987.1	0.0	0.2	8.0	1.1	TTGAAGATGTTCTATTTCTGT
Nta-miR23	22	ET679542.1	58.5	62.3	0.4	0.2	TGGTAGACGTAGGATTTGAAGA
Nta-miR24a	21	FH052337.1	0.2	1.3	0.1	0.1	AAAATGTGGCCGGATACGTGT
Nta-miR24b	21	FH324277.1	0.2	1.3	0.1	0.1	AAAATGTGGCCGGATACGTGT
Nta-miR25	22	FH081190.1	2.6	20.2	389.8	12.9	TGAACTCTCTCCCTCAATGGCT
Nta-miR26a	21	ET837426.1	35.1	179.9	2.2	0.4	ATTGTTACATGTAACACTGGC
Nta-miR26b	21	FH393496.1	483.9	1146.6	1.5	1.3	ATTGTTACATGTAGCACTGGC
Nta-miR27a	22	FH905151.1	0.7	1.3	0.2	0.1	AAGTTCGATTTGTACGAAGGGC
Nta-miR27b	22	FH915892.1	0.7	1.3	0.2	0.1	AAGTTCGATTTGTACGAAGGGC
Nta-miR27c	22	FH918314.1	0.7	1.3	0.2	0.1	AAGTTCGATTTGTACGAAGGGC
Nta-miR28	22	FH253762.1	11.4	11.4	13.4	12.3	TAGCATAGAATTCTCGCACCTA
Nta-miR29	21	FH324206.1	0.0	2.1	0.0	0.0	ACGGGTGCGGCTACATTTTGG
Nta-miR30	21	FI086539.1	16.6	7.7	2.2	0.2	ATCGTAACATATAGCACTAGC
Nta-miR31	24	ET823610.1	0.0	0.0	0.9	0.6	GCATATATGGGCCAACTGTGTAAC
Nta-miR32	21	FH560937.1	0.0	0.0	1.6	1.7	TGAACTCCAGCATATTATACT
Nta-miR33a	24	FH761979.1	0.2	0.4	4.0	5.3	GCTGGACCGGTATACTTTGCTGAC
Nta-miR33b	24	FH762383.1	0.2	0.4	4.0	5.3	GCTGGACCGGTATACTTTGCTGAC
Nta-miR34	22	ET840416.1	1.6	24.7	13.2	7.8	TTCCCGACTCCCCCCATACCAC
Nta-miR35	21	FH119649.1	0.0	0.0	5.4	1.1	AGAAAAATGGTAGCCATTGGA
Nta-miR36	24	FH408012.1	0.0	0.0	21.6	27.3	AATATACTGGAGTTCGGTGCACCT
Nta-miR37	22	FH666318.1	0.0	0.0	3.3	0.4	TGGAAGTACTGCCTAAGTTTGA
Nta-miR38a	21	FH681839.1	0.8	1.0	0.5	0.1	TCACATAAATTGAAACGGAGG
Nta-miR38b	21	FH968988.1	0.8	1.0	0.5	0.1	TCACATAAATTGAAACGGAGG

See Additional file 1: Table S1 for the detailed pre-miRNA sequences and their secondary structures

#### Identification of loci generating phased small RNAs

**Figure 2:**
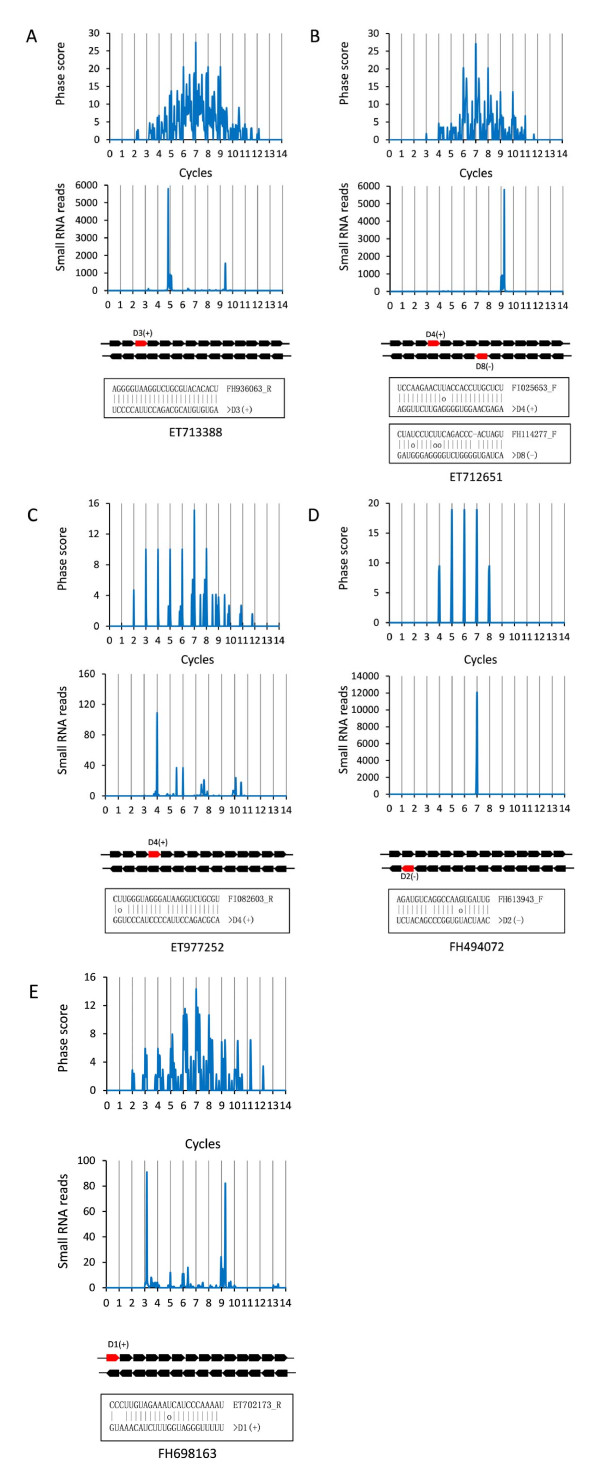
**Examples of loci generating phased 21-nt small RNAs (FH494072 and FH698163) and phased 24-nt small RNAs (ET713388, ET712651 and ET977252)**. The middle and the top graph in each locus represent the distribution of small RNA reads across the whole locus and the phase signals generated from the small RNA reads, respectively. Each arrow underneath the plot graph represents a 21-nt or 24-nt phase with the arrow direction indicating the forward or the reverse strand. The red arrows represent the phase with the highest number of small RNA reads. Target(s) of the phased small RNA corresponding to the red arrow, identified by degradome sequencing, are shown at the bottom of each graph.

To identify loci generating phased small RNAs, small RNA clusters were first defined based on the number of small RNAs aligned to a certain length of sequence segment and the relative distance between neighbouring small RNAs [24]. Using the criteria mentioned in Methods, we identified 1,890-15,252 21-nt clusters and 11,726-107,765 24-nt clusters in four samples (Table 3). As expected, there were more 24-nt clusters than 21-nt clusters. These small RNA clusters were then analysed using the methodology described previously to see whether the small RNAs within these clusters distributed in a phased pattern, which was determined by a phase score[42]. A higher phase score indicates a stronger phase signal. With a phase score of 1.4 as suggested previously [42], we found that 189-3,098 and 1,359-20,112 loci were able to produce 21-nt and 24-nt phased small RNAs in four samples, respectively. The number of loci generating phased small RNAs was significantly reduced when the phase score was increased to 10 (Table 3). These loci are shown in Additional file 2: Table S2, and five representative loci generating 21-nt- or 24-nt-phased small RNAs are shown in Figure 2.

**Table 3:** Number of small RNA clusters and loci generating 21-nt- or 24-nt-phased siRNAs

			Root		Leaf
		
Category	Phase size (nt)	Control	Topping	Wounding	Wounding
Small RNA clusters	21	1890	5116	15252	6559
	24	11726	37000	107765	84270
Phased siRNA generating loci (*P > *1.4)	21	189	919	3098	973
	24	1359	5244	20112	14681
Phased siRNA generating loci (*P *> 10)	21	13	50	220	57
	24	7	115	691	508

Loci homologous to known house-keeping noncoding RNAs, such as rRNA, tRNA, snRNA and snoRNA and overlapped with repeat sequences were excluded. The phase scores (*P *values) were calculated following the description in Howell et al. [42]

In plants, the typical loci generating phased siRNAs are *TASs *[20-22]. *TAS3 *with the miR390 cleavage sites has been found to be conserved in plants, including tobacco. In this study, we experimentally confirmed the conservation of *TAS3 *in tobacco. At least one *TAS3 *gene (GSS acc. no. FH434354) with two miR390 binding sites and generating the conserved ta-siARFs was confidently identified with a phase score of 31.3. We searched targeting sites of the newly identified miRNAs in this study in loci with a phase score over 10, and found several such loci were potentially targeted by Nta-miR15, Nta-miR22, Nta-miR31, Nta-miR33, Nta-miR36 or Nta-miR37(Additional file 2: Table S2). Whether these novel miRNAs are involved in biogenesis of the phased small RNAs observed is waiting to be investigated experimentally.

### Identification of small RNA targets by degradome sequencing

#### Targets of miRNAs

Recently, degradome sequencing has been successfully applied to identify targets of miRNAs in *Arabidopsis *and rice [43,44]. Taking the advantage of this new approach, we sequenced a degradome library constructed using total RNA isolated from roots of leaf-wounding treated tobacco plants. Over 14 million of raw reads were generated. Out of 685,411 unique clean reads (excluding reads with low quality and mapped to known house-keeping noncoding RNAs), representing ~11.8 million of raw reads, 55.2% (378,597) and 56.5% (387,518) were mapped to tobacco ESTs and GSSs, respectively. These mappable reads were then used for target gene identification using the CleaveLand pipeline as described [43,45].

**Figure 3:**
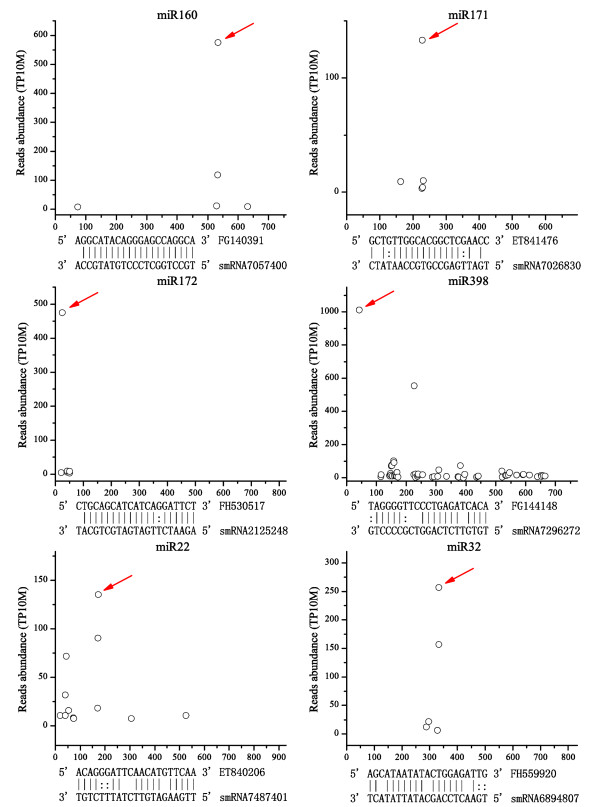
**Plot signals of the candidate targets of miRNAs predicted by degradome sequencing**. Targets of conserved miRNAs (miR160, miR171, miR172 and miR398) and tobacco-specific miRNAs (Nta-miR22 and Nta-miR32) are shown. The X-axis displays the nucleotide position of the target genes. The Y-axis indicates the abundance of reads converted into transcripts per 10 million (TP10M). Each circle represents a degradome fragment mapped to the target gene and the circle indicated by the red arrow represents the expected miRNA cleavage product.

One or more targets were uncovered for 12 conserved and five tobacco-specific miRNA families (Additional file 3: Table S3). These targets were classified into five categories following the description indicated in the software CleaveLand 2. Six representative miRNA targets identified by degradome sequencing were shown in Figure 3. Fourteen targets of seven miRNA families were fallen into Category 0 and 1, for which the most abundant cleavage signal was detected at the expected miRNA-mediated cleavage sites. Functions of the conserved miRNA target genes are evolutionary conserved between different plants, including tobacco. For example, miR160 has been shown to regulate expression of *ARF *(auxin-response factor) genes in *Arabidopsis *and tomato [46,47]. Our results showed that *ARF *is one of the potential targets of miR160 in tobacco, suggesting a conserved role of miR160 in tobacco. Similarly, conserved targets were also identified for miR171 (hairy meristem gene, a *SCARECROW-like *transcription factor) and miR172 (*AP2*-domain containing genes) (Additional file 3: Table S3). However, conserved miRNAs might have non-conserved function in tobacco, for instance, *salicylic acid-induced protein 19*, which has been reported to have multiple functions in defense response in tobacco [48], was the only target found for miR164, suggesting that miR164 could be part of the defensive pathway involving the salicylic acid signal.

The newly identified miRNAs were found to have diverse targets, but most of them seem to have a role in stress responses, suggesting that most of the newly identified miRNAs could function as regulators of defensive pathways in tobacco. For example, our degradome sequencing result showed that Nta-miR10 targets a gene encoding Avr9/Cf-9 rapidly elicited protein 4 (ACRE4), which plays a role in resistance to wounding and mechanical stress in tobacco [49]. Nta-miR38 targets genes encoding nematode resistance-like protein and ankyrin repeat containing protein, both are induced upon infection of disease [50,51].

#### Targets of phased small RNAs

One of the phased small RNAs generated from the *TAS3 *locus is ta-siARF, which targets *ARF *genes that influence leaf morphology and lateral root growth [52,53]. To know whether the phased small RNAs identified in this study, like ta-siARF, are able to regulate expression of other genes, we checked the presence of potential targets of the most abundant 30 phased small RNAs observed in the damage-treated samples in our degradome sequencing dataset. We found targets for five (two 21-nt and three 24-nt) of the 30 selected phased small RNAs (Figure 2; Additional file 4: Table S4). For example, the D2(-) phased small RNA from locus FH494072, which was specifically detected in wounding or topping treated samples, was found to target an hypothetical protein; the D4(+) phased small RNA from locus ET712651 was found to target several genes with diverse functions, including putative disease resistance protein, dicer-like protein and type-A phytochrome.

**Figure 4:**
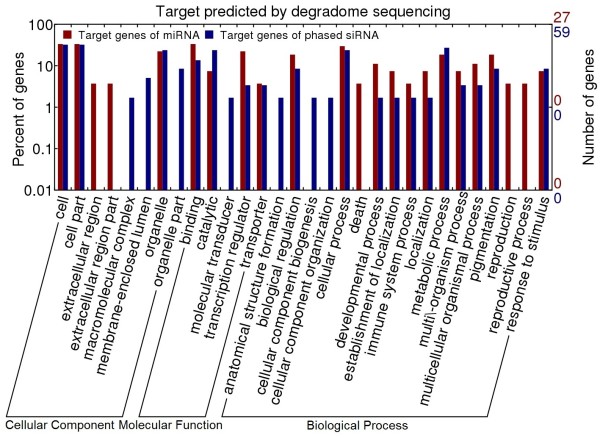
**Gene ontology analysis of the targets of miRNAs and the phased small RNAs**.

We did gene ontology (GO) analysis for the target genes of miRNAs and phased small RNAs. Although miRNAs and phased small RNAs had most of their targets grouped into the same GO terms, including response to stimulus and immune system process, it seems that miRNAs and the phased small RNAs are targeting specific genes involved in reproduction (for miRNAs) and cellular component biogenesis and organization (for the phased small RNAs), respectively (Figure 4), suggesting a unique role of miRNAs and the pahsed small RNAs in these processes.

### Small RNAs responsive to topping and wounding treatments

#### Topping and wounding responsive miRNAs

**Figure 5:**
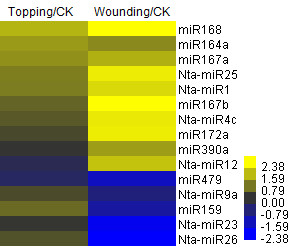
**Heat map showing miRNAs with a significant expression change upon wounding or topping treatment in roots**.

In order to compare expression levels of small RNAs in different samples, the abundance of each unique small RNA was normalized by converting the absolute read numbers to relative read numbers (reads per million) based on the total number of reads in each library. To get insight into the potential role of miRNAs in response to damage treatments, we compared the expression changes of all miRNAs detected in our datasets. Most of the miRNAs identified in this study changed their expression upon topping or wounding treatment. Eight conserved and seven newly identified miRNAs showed significant up-regulation or down-regulation upon damage treatment (Figure 5). Out of the eight conserved miRNAs, miR159, miR164, miR167 and miR172 have been previously shown to be stress-responsive [15]. miR164 and miR168 were significantly induced in roots by both topping and wounding treatments. miR172 and miR390 were significantly induced only in roots treated by wounding. Furthermore, miR159, miR319 and miR2911 were up-regulated only in topping treated sample while were down-regulated or remaining unchanged in wounding treated sample. These results indicate different miRNA families behave differently upon damage treatment, suggesting a different role of these miRNAs in response to damage treatment in tobacco. In addition, seven conserved miRNA families (miR169, miR395, miR397, miR398, miR399, miR408 and miR827) were not detected in the untreated tobacco root sample but were detected in at least one damage treated sample (Additional file 1: Table S1). Among these miRNAs, miR395 and miR398 have been shown to response to a variety of stresses [15].

**Figure 6:**
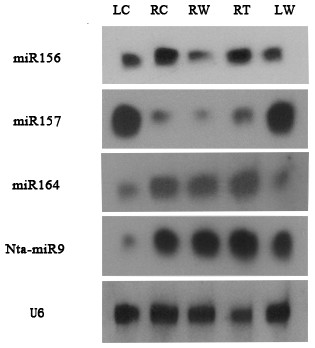
**Northern blot analysis of miRNA expression**. LC: control leaves; RC: control roots; RW: roots from leaf wounded plants; RT: roots from topping treated plants; LW: leaves from leaf wounded plants. Tobacco U6 was served as a loading control.

To confirm the deep sequencing results, the expression levels of miR156a, miR157 and miR164a were analysed using northern blot. According to northern results, the expression levels of miR164a and miR156a were higher in roots than in leaves, while the expression levels of miR157 were higher in leaves than in roots (Figure 6), consistent with the deep sequencing results (Additional file 1: Table S1); however, for miR164a, the induction detected by northern analysis was not as significant as that detected by deep sequencing; for miR156a and miR157, a discrepant result was observed in the wounding treated root samples between deep sequencing and northern analysis (Figure 6; Additional file 1: Table S1). The induction observed in deep sequencing results could be an artifact caused by biased adaptor ligation and PCR amplification during the procedure of small RNA library construction.

Out of the 38 newly identified miRNA families, 27 were relatively highly expressed, particularly Nta-miR9, Nta-miR17 and Nta-miR26 (Table 2). Northern analysis of Nta-miR9 showed that this novel miRNA is highly expressed in roots but lowly expressed in leaf, consistent with the deep sequencing results. Nta-miR9 was highly induced by wounding in leaves and slightly induced in roots from the topping treated plants (Figure 6), but a reduction of this miRNA in roots from the wounding treated plants detected by deep sequencing was not detected by northern analysis, which could be because of cross hybridization of other members of this miRNA family that failed to be detected by deep sequencing. The discrepancy observed between deep sequencing and northern blot analysis suggests that caution must be taken when directly compare small RNA expression levels observed by either approach.

Nta-miR1, Nta-miR4c and Nta-miR25 were highly induced in roots by both wounding and topping treatments; Nta-miR26 was highly induced only in topping treated sample (Figure 5). Nine newly identified miRNA families (Nta-miR15, Nta-miR21-22, Nta-miR29, Nta-miR31-32 and Nta-miR35-37) were not expressed in the untreated roots but were detected in damage treated samples. Different members of the same miRNA family could behave differently upon damage treatment, for example, Nta-miR4a.2 was highly induced by damage treatment, whereas Nta-miR4a.1 and miR4b were not induced.

#### Topping and wounding responsive phased small RNAs

**Figure 7:**
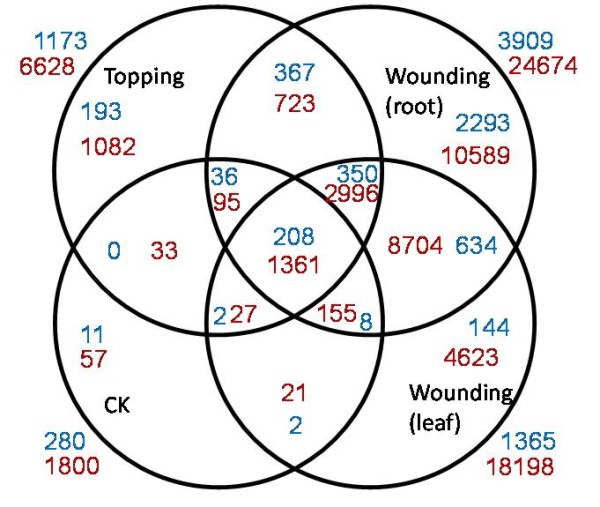
**Venn diagram showing the number of loci (with a phase score over 1**.4) generating phased small RNAs in four libraries. Blue and red numbers represent the number of loci generating 21-nt- and 24-nt-phased small RNAs, respectively.

Generally, the number of small RNA clusters was much higher in the damage treated samples (Table 3), indicating a number of small RNAs could be induced by these treatments. The number of loci generating both 21-nt- and 24-nt-phased small RNAs increased dramatically after topping or leaf-wounding treatment. Some of these loci were found in two, three or all four samples, but some of them were unique to each treatment (Figure 7). Similar to what has been found for miRNAs, wounding treated root sample had the most abundant uniquely up-regulated loci that generate phased small RNAs (Figure 7). Two phased small RNAs were analysed using northern blot, but no signal was detected (data not shown), mainly due to their low expression levels.

## Discussion

Recent computational prediction identified 259 potential conserved tobacco miRNAs of 65 families [39], but computational prediction based on known miRNAs is unable to identify novel miRNAs and miRNAs expressed under specific conditions, such as biotic and abiotic stresses. In this study, we used high-throughput sequencing approach to experimentally confirm expression of the known miRNAs and their changes upon wounding or topping treatment, and to identify novel tobacco-specific miRNAs. Our study detected the expression of 100 known miRNAs (belonging to 27 families) in tobacco roots and leaves, including miR408, miR477, miR1919, miR2118 and miR2911 that was not previously computationally identified. More importantly, our work identified 59 novel tobacco-specific miRNAs (belonging to 38 families).

Upon damage treatments, eight conserved and seven newly identified miRNAs showed significant up-regulation or down-regulation (Figure 5). In addition, seven conserved and nine newly identified miRNA families were only detected in the damage treated sample(s). The majority of these damage-responsive conserved miRNA families have been previously shown to be stress responsive in other plant species, suggesting that these miRNAs might also play a positive regulatory role in stress-tolerance in tobacco. In *Arabidopsis*, miR164 is essential for floral, leaf and root development [54,55]. We found that this miRNA was induced in roots upon wounding or topping treatment (Table 2; Figure 6) and that one of the targets encodes a salicylic acid-induced protein; therefore, it is possible that miR164 is required for homeostasis of defensive proteins in tobacco. In addition, most targets of the newly identified miRNAs responsive to topping or wounding, found in the degradome sequencing results, seem to be regulators of various defensive pathways, implicating the importance of these miRNAs in stress responses in tobacco.

Different damages seem to have different effects on the expression of miRNAs and siRNAs. The abundance of small RNAs in the wounding treated leaf sample was generally much higher than that in the topping and wounding treated root samples, suggesting that the induction of small RNAs is more significant in the tissue where the damage was directly applied. Induction of small RNAs in roots collected from the leaf-wounding plants suggests that a mobile signal responsible for this induction is generated in leaves and transported to roots. This signal is most likely a type of phytohormone, such as salicylic acid, jasmonic acid or ethylene. However the exact nature of this signal is waiting to be resolved.

Silencing of RNA-directed RNA polymerase 1 (RdR1) makes *Nicotiana attenuata *highly susceptible to insect herbivores, suggesting the defense elicitation in *Nicotiana attenuata *is under the direct control of small RNAs [56]. Further investigation has found large-scale changes of small RNAs associated with insect elicitation [57]. Wounding is similar to insect herbivores and induction of small RNAs in the wounding treated tobacco plants is consistent with this recent finding. This result suggests that small RNAs could play a central role in reprogramming the tobacco transcriptome in response to biotic and abiotic stresses. It is unclear how the stress-transcriptome of tobacco has changed upon wounding or topping, but our work has provided bases for further investigation on roles of small RNAs in stress responses in tobacco.

In plants, *TASs *are the noncoding transcripts targeted by miRNAs to generate phased small RNAs. Recent investigation has demonstrated that the 22-nt miRNAs, rather than the 21-nt miRNAs, are the triggers for production of secondary phased small RNAs [41]. Out of the 38 newly identified miRNA families, 13 families have a 22-nt miRNA. In addition, we found that a large number of genomic regions in tobacco are able to produce phased 21-nt or 24-nt small RNAs and that a number of these regions containing potential binding sites of the newly identified miRNAs. Although we did not find these binding sites are enriched for 22-nt miRNAs, it is worth to perform further detailed analysis to see if the 22-nt miRNAs found in this study play a role in biogenesis of the secondary phased small RNAs.

## Conclusions

Our study experimentally confirmed the conservation of a number of known miRNAs in tobacco, including conserved miRNAs that were not previously identified by computational prediction. Identification of damage-responsive miRNAs and phased small RNAs and defense related targets of these small RNAs suggest that the inducible defense responses in tobacco might be controlled by pathways involving small RNAs.

## Methods

### Plant materials and sample preparation

All tissue samples were collected from tobacco cultivar, Hicks Broad Leaf. Plants were grown in the growth room with a temperature of 22-25°C in order to minimize external affects on the biosynthesis of nicotine alkaloids. Three 40-day-old plants with roughly identical size were selected for the experiment. One was used for mechanical leaf wounding by making punctures on at least five fully expanded leaves, one was used for the topping treatment, and the third one as control (CK). After damage treatments, plants were kept for another 48 hours in the growth room before sample collection to allow occurring of damage induced responses. Roots were collected from the wounding and topping treated plants as well as the control plant. Leaf samples were collected from the wounding treated and control plants.

### RNA isolation, small RNA and degradome sequencing

Tissues were ground into a fine powder in liquid nitrogen and total RNAs were extracted using the TRIzol^® ^Reagent kit (Invitrogen). Four small RNA libraries (leaves and roots from the wounding treated plant, roots from the topping treated and the control plants) were constructed using the Small RNA Sample Prep Kit (Illumina) [58,59]. Briefly, small RNAs were fractionated on the 15% polyacrylamide, 8 M urea gel. The 18-25 nt portion of the gel was excised and small RNAs were eluted and purified using the nucleic acid purification kit (Axygen). Small RNAs were then sequentially ligated with a 5' RNA adapter (5'-GUUCAGAGUUCUACAGUCCGACGAUC-3') and a 3' RNA adapter (5'-pUCGUAUGCCGUCUUCUGCUUGUidT-3'). cDNA libraries were constructed through reverse transcription using SuperScript™ III (Invitrogen) and enriched by 15 cycles of PCR. The final PCR products were then purified using the PureLink™ PCR Purification Kit (Invitrogen) and sequenced using the SOLEXA genome analysis system. Primers used were: 5'-CAAGCAGAAGACGGCATACGA-3' (RT primer), 5'- CAAGCAGAAGACGGCATACGA-3' (small RNA PCR primer 1), 5'-AATGATACGGCGACCACCGACAGGTTCAGAGTTCTACAGTCCGA-3' (small RNA PCR primer 2) and 5'-CGACAGGTTCAGAGTTCTACAGTCCGACGATC-3' (small RNA sequencing primer).

The degradome library was constructed according to a published protocol [60]. Briefly, RNA fragments with a poly(A) tail were isolated from total RNA of wounding treated leaves using the Oligotex mRNA mini kit (Qiagen), and then a 5' RNA adapter with a *Mme*I restriction site at its 3' end was added to the 5' ends of the isolated poly(A) RNAs. After reverse transcription using oligo d(T) and PCR enrichment, the PCR products were purified and digested with *Mme*I. After ligating a double-stranded DNA adapter to the 3' end of the digested products, the ligated products were further purified and amplified, and then sequenced using the Illumina GA II platform.

### Tobacco sequences and other databases

The tobacco reference genomic (1,420,578 GSSs) and EST (80,783 generated from tobacco cultivar Hicks Broad Leaf by TGI) sequences were retrieved from GenBank. The nt database were downloaded from GenBank for annotation of the candidate miRNA genes and phased siRNA loci. Rfam 9.1 (http://rfam.janelia.org/) and RepBase 14.03 (http://www.girinst.org) were used for filtering known RNAs and repeat sequences. Transcription factor sequences of tobacco were retrieved from http://compsysbio.achs.virginia.edu/tobfac/[61]. Genes involved in nicotine pathways were collected from previous studies [26-32]. miRNAs in miRBase 16.0 were used for identification of tobacco homologs of the known miRNAs (http://www.mirbase.org/).

### miRNA identification

All small RNA data were processed by a suite of perl scripts. To identify candidate miRNAs, a pair of small RNAs located at the same GSS or EST and no more than 400-nt apart were selected and the sequence between these two small RNAs was subjected to prediction of stem-loop structure using the Vienna RNA package [62]. Candidates (pre-miRNAs) with an ideal hairpin structure containing the pair of small RNAs that is able to form a miRNA::miRNA* duplex with less than 4 mismatches and 2-nt of 3' overhangs were selected. Small RNAs aligned to these selected candidates were checked to eliminate the candidates with a smear distribution pattern of the small RNAs. For a certain miRNA family, candidates with any mismatch in mature miRNAs or more than 3 mismatches in pre-miRNAs were treated as different members. Redundant pre-miRNA sequences were eliminated.

### Identification of loci generating phased small RNAs

The locations of small RNA clusters were pinpointed with the definition of a small RNAs cluster as a segment of sequence mapped by at least 9 distinct small RNAs and without other small RNAs in its flanking 100-bp regions [42]. A pipeline containing several perl scripts were then employed to identify loci generating phased siRNAs from the identified small RNA clusters [24].

### Target identification by degradome sequencing

After adaptor trimming and filtering out the reads mapped to house-keeping noncoding RNAs, such as tRNAs, rRNAs, snRNAs and snoRNAs, 20-21-nt high quality reads were kept for further analysis. Targets of miRNAs and phased small RNAs were identified using the CleaveLand pipeline [45]. Briefly, 20-21-nt small RNA reads were first mapped to tobacco ESTs and GSSs separately with the SHRiMP program. The reference sequence fragments of 30 nt were then spliced to make sure the position with degradome reads mapped was included. Fragments were further analyzed to see if they are targeted by miRNAs. The fragments with an alignment cutoff value less than 7 were considered as candidate targets. The target genes identified by degradome sequencing can be classified into five categories based on the relative abundance of degradome transcripts mapped to the expected cleavage sites and other sites.

### Northern blot analysis of miRNA expression

Total RNA was isolated from tobacco leaves and roots collected from plants treated with wounding or topping or untreated (control) using the TRIzol^® ^Reagent kit (Invitrogen). Approximate 20 μg of total RNA was fractionated on a 15% denaturing gel for 2 hours and then transferred electrophoretically to the nylon membrane (Nytran SuperCharge membranes). Membranes were then UV cross-linked in a Straralinker 1800 Stratagene and baked for at least 2 hours at 80°C. DNA oligos complementary to miRNA sequences and labeled with digoxin were synthesized by Invitrogen. Membranes were prehybridized for 2 hours at 50°C using PerfectHyb™ Plus buffer (SIGMA) and then hybridized with digoxin-labeled probes for 16-20 hours at 42°C. Membranes were then washed three times (two times with 2 × SSC + 0.1%SDS and one time with 0.5 × SSC + 0.1%SDS) and sealed with the Blocking Solution (SIGMA) containing Anti-Digoxigenin-AP (Roche) for 1 hour. Membranes were finally incubated with Detection Buffer (0.1 M Tris-Cl, 0.1 M NaCl, pH9.5) and CSPD (Roche) for 5 min and exposed to X-ray films for 4 hours or overnight.

## Authors' contributions

LF, QHZ and JX conceived the experiments. LF and ST designed the experiments. ST performed the experimental work. YW did the small RNA analysis. ZL carried out the degradome data analysis. YG helped in RNA isolation. BX provided plant materials. ST, QHZ and LF wrote the paper. All authors read and approved the final manuscript.

## Supplementary Material

Additional file 1 **Table S1**. Tobacco miRNA families identified in this study. Click here for file

Additional file 2 **Table S2**. Loci with significant (phase score > 10) 21-nt or 24-nt phase signals. Click here for file

Additional file 3 **Table S3**. miRNA targets identified by degradome sequencing. Click here for file

Additional file 4 **Table S4**. Identification of targets of siRNAs from the phased-siRNA generating loci by degradome sequencing. Click here for file
